# Invasive Saprochaete capitata Infection in an Immunocompromised Patient With Acute Myeloid Leukemia: A Case Report

**DOI:** 10.7759/cureus.85236

**Published:** 2025-06-02

**Authors:** Siham Karrati, Najmeddine Kharbouch, Awatif El Hakkouni

**Affiliations:** 1 Parasitology-Mycology Department, Mohammed VI University Hospital, Cadi Ayyad University, Marrakech, MAR

**Keywords:** acute myeloid leukemia, antifungals, hematological malignancies, invasive fungal infection, neutropenic patient, saprochaete capitata

## Abstract

*Saprochaete capitata* (*S. capitata*) is a rare but emerging opportunistic fungal pathogen, identified as an arthroconidial yeast-like filamentous fungus. It can cause potentially life-threatening invasive fungal infections (IFIs) in immunocompromised patients, particularly those with hematological malignancies and profound neutropenia, and is associated with poor clinical outcomes. Diagnosing invasive *S. capitata* infections is challenging, relying primarily on clinical suspicion and isolation of the pathogen from blood, other sterile body fluids, or tissue biopsies. Due to its resistance to both echinocandins and fluconazole, *S. capitata* presents significant treatment challenges, with no established optimal therapeutic strategy for invasive infections.

Here, we present a case of a 46-year-old man with acute myeloid leukemia who developed an invasive *S. capitata* infection with fungemia and pulmonary involvement during post-chemotherapy aplasia. Despite profound immunocompromise, the patient successfully recovered following treatment with combination antifungal therapy, which included liposomal amphotericin B and voriconazole.

This case highlights the critical importance of early diagnosis and prompt initiation of appropriate antifungal therapy, particularly in immunocompromised patients, to reduce the exceptionally high mortality and morbidity associated with this severe IFI.

## Introduction

*Saprochaete capitata* (previously named *Geotrichum capitatum*, *Blastoschizomyces capitatus*, and *Magnusiomyces* *capitatus*) is an arthroconidial yeast-like filamentous fungus belonging to the *Ascomycetes* clade, within the class *Saccharomycetes*, the order *Saccharomycetales*, and the family *Dipodascaceae* [1,2]. This yeast is commonly found in environmental sources such as soil, water, air, plants, and dairy products. It is also known to be a part of the normal flora of the human skin, gastrointestinal system, and respiratory tract [1,3]. However, it is primarily an opportunistic pathogen, causing invasive infections in immunocompromised patients, particularly those with hematological malignancies such as acute leukemia, especially in the presence of profound neutropenia [1,3-5].

The clinical features of invasive *S. capitata* infections are often nonspecific and may be misinterpreted as invasive candidiasis, with fungemia commonly observed. However, *S. capitata* more frequently involves the lungs and other deep organs compared to* Candida *spp. [1,5,6]. The diagnosis is challenging, requiring a high index of clinical suspicion and isolation of the pathogen from blood, other sterile body fluids, or tissue biopsies [1]. *S. capitata* presents significant therapeutic challenges due to its intrinsic resistance to echinocandins and high resistance to fluconazole [1,5]. As a result, no optimal therapeutic strategy has been established for treating *S. capitata* infections. Clinical management often involves the use of combination antifungal therapies, with careful consideration of the organism's susceptibility to other antifungal agents [5]. Despite appropriate antifungal therapy, invasive *S. capitata* infections are associated with a poor prognosis, with a mortality rate exceeding 50% [4].

Although still considered rare, invasive infections caused by *S. capitata* have shown an increasing trend in recent years, particularly among patients with hematologic malignancies. The organism has been implicated in several hospital outbreaks across Europe, with Italy reporting the highest number of cases [1]. This increasing recognition highlights* S. capitata* as an emerging opportunistic pathogen with significant clinical implications in immunocompromised hosts [1,3,5]. Given the paucity of large-scale epidemiological data and the absence of evidence-based consensus on optimal diagnostic and therapeutic approaches, current clinical management is largely guided by limited case series and individual case reports [1]. Therefore, detailed case reports are invaluable for enhancing pathogen awareness, elucidating clinical presentation, and informing evidence-based management strategies.

Here, we report a case of invasive *S. capitata* infection with fungemia and pulmonary involvement in a severely immunocompromised patient with acute myeloid leukemia (AML) during post-chemotherapy aplasia. The patient was successfully treated with combination antifungal therapy, including liposomal amphotericin B and voriconazole.

## Case presentation

A 46-year-old man was admitted to the hematology department in October 2024 for the treatment of AML. He received induction therapy following the "7 + 3" protocol, which includes a combination of cytarabine for seven days and daunorubicin for three days, both administered via continuous intravenous infusion. On day 12 of chemotherapy, during bone marrow aplasia, the patient developed a high-grade fever reaching 40°C. On day 13, he reported a new onset of respiratory symptoms, including dyspnea and dry cough.

On physical examination, the patient was conscious, febrile at 40°C, with a blood pressure of 130/70 mmHg, a pulse rate of 94 beats per minute, a respiratory rate of 30 breaths per minute, and an oxygen saturation of 90%. Pulmonary examination revealed bilateral crackles. Other clinical examinations were unremarkable.

Multiple blood cultures were drawn during febrile peaks, and the patient was started empirically on injectable cefepime (2 g three times daily).

Laboratory investigations revealed the following results: a white blood cell count of 380 cells/mm³ with an absolute neutrophil count of 20 cells/mm³, a hemoglobin level of 6.3 g/dL, and a platelet count of 21,000 cells/mm³. Inflammatory markers were elevated, with an erythrocyte sedimentation rate of 90 mm/h and a C-reactive protein level of 104 mg/L.

Given the persistent fever in the setting of profound neutropenia and new-onset respiratory symptoms, an invasive fungal infection was suspected.

**Figure 1:**
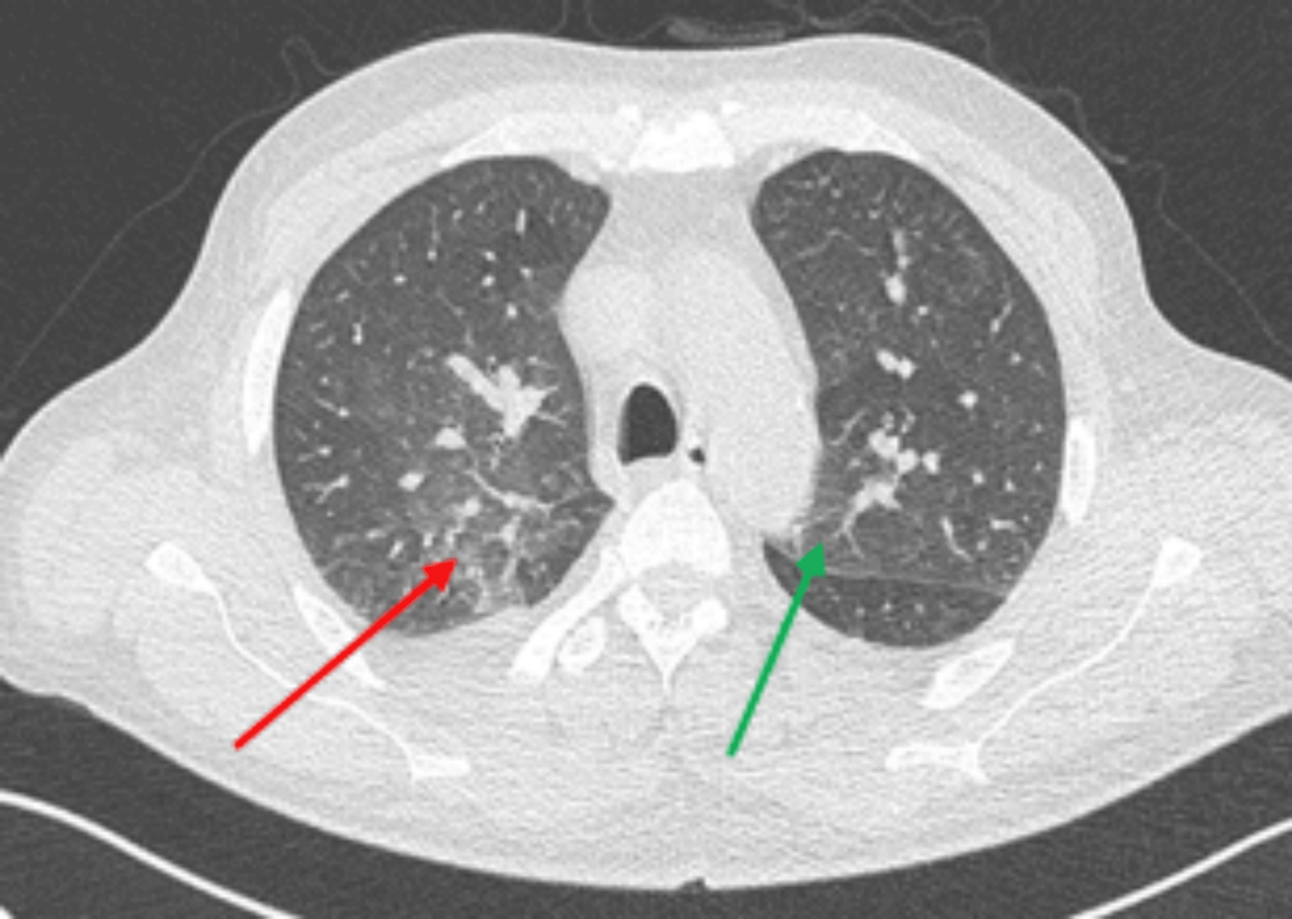
Chest CT scan showing ground-glass opacities in the apicodorsal segment of the right upper lobe (red arrow) and the lingula (green arrow).

A chest computed tomography scan was performed, revealing bilateral ground-glass opacities, more pronounced on the right lung (Figure 1). Routine bacterial cultures and respiratory virus testing using the FilmArray respiratory panel with multiplex polymerase chain reaction (PCR) yielded negative results. Galactomannan antigenemia was also negative. The patient was scheduled for a bronchoscopy to perform a bronchoalveolar lavage (BAL).

**Figure 2:**
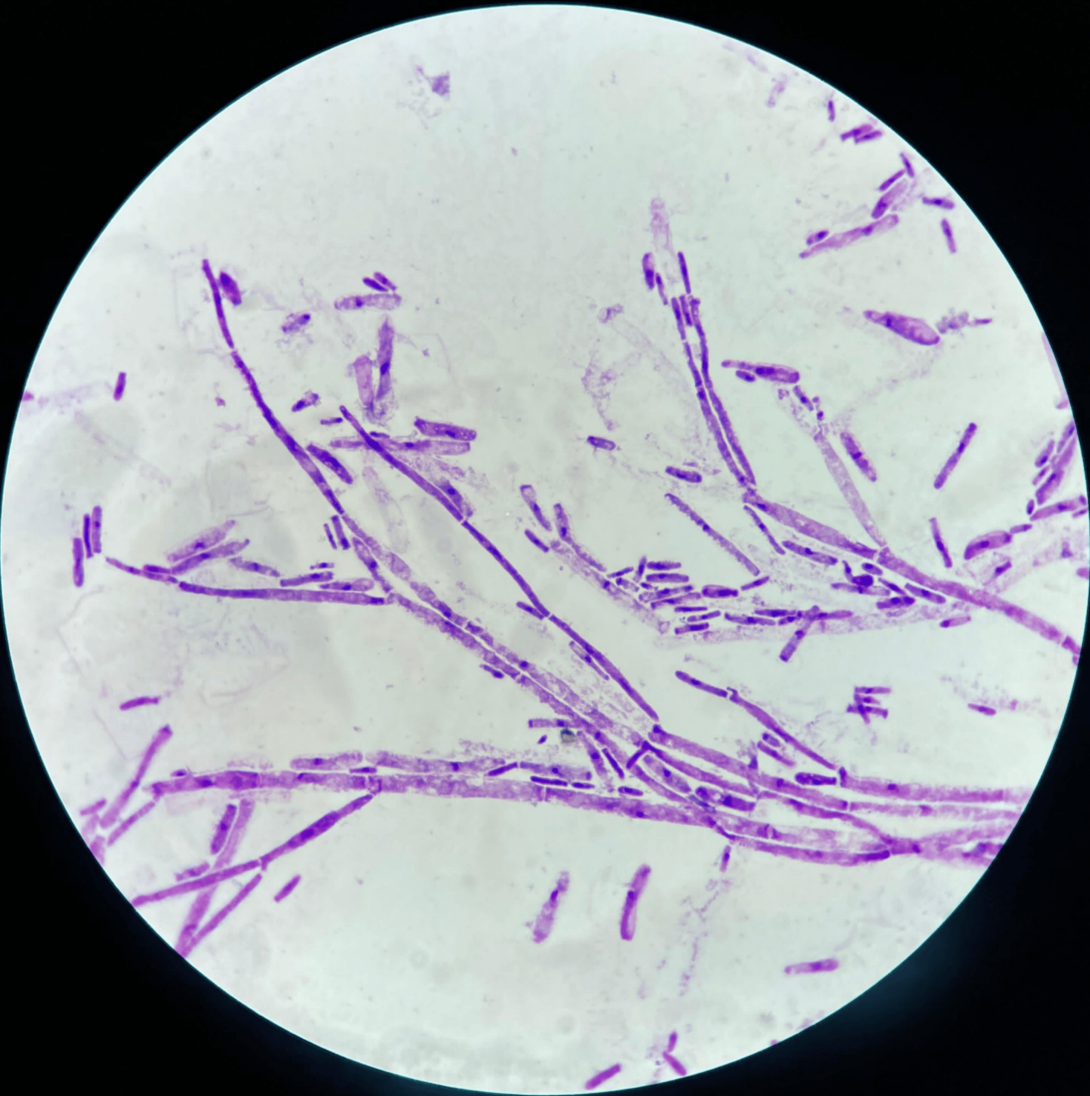
Gram-stained blood culture smear showing septate hyphae and arthroconidia (1000× magnification).

**Figure 3:**
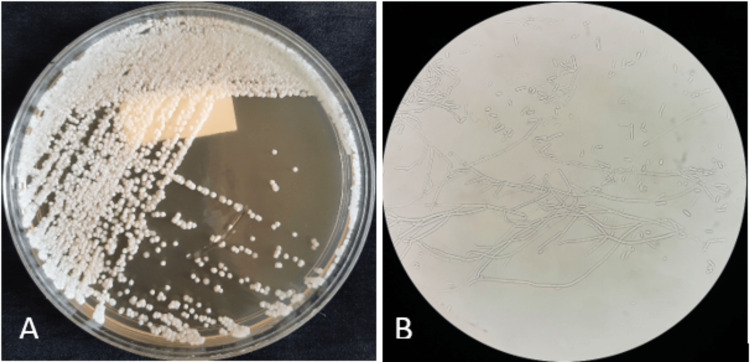
(A) Growth of white, dry, cottony, wrinkled, and circular colonies of Saprochaete capitata after 24 hours of incubation at 35°C on Sabouraud chloramphenicol agar. (B) Wet mount preparation of blood culture showing septate, hyaline hyphae branching at acute angles and rectangular arthroconidia (400× magnification).

On the second day of febrile neutropenia, two blood cultures were positive after 24 hours of incubation in the BD BACTEC system (BD, Franklin Lakes, NJ). Direct examination after Gram staining revealed septate hyphae and arthroconidia (Figure 2). The patient was immediately started on intravenous liposomal amphotericin B (5 mg/kg/day) pending the results of antifungal susceptibility testing. The culture on Sabouraud chloramphenicol agar showed the growth of numerous white-colored, dry, cottony, wrinkled, and circular colonies after 24 hours of incubation at 35°C (Figure 3A). Wet mount preparation of the culture revealed rectangular arthroconidia and septate hyaline hyphae branched at acute angles (Figure 3B). The isolated colonies were processed using the VITEK 2 Compact (bioMérieux, Marcy-l'Étoile, France) with a YST (yeast identification) card, which identified the yeast as *S. capitata* with a confidence value of 98%. The strain was sensitive to amphotericin B with a minimum inhibitory concentration (MIC) of 0.5 μg/mL, to voriconazole with a MIC ≤ 0.12 μg/mL, and to flucytosine with a MIC ≤ 2 μg/mL. High MICs were observed for fluconazole (16 μg/mL), caspofungin (32 μg/mL), and micafungin (32 μg/mL). Based on these results, antifungal treatment was escalated with the administration of voriconazole (6 mg/kg twice a day as a loading dose, followed by a maintenance dose of 4 mg/kg twice a day).

**Figure 4:**
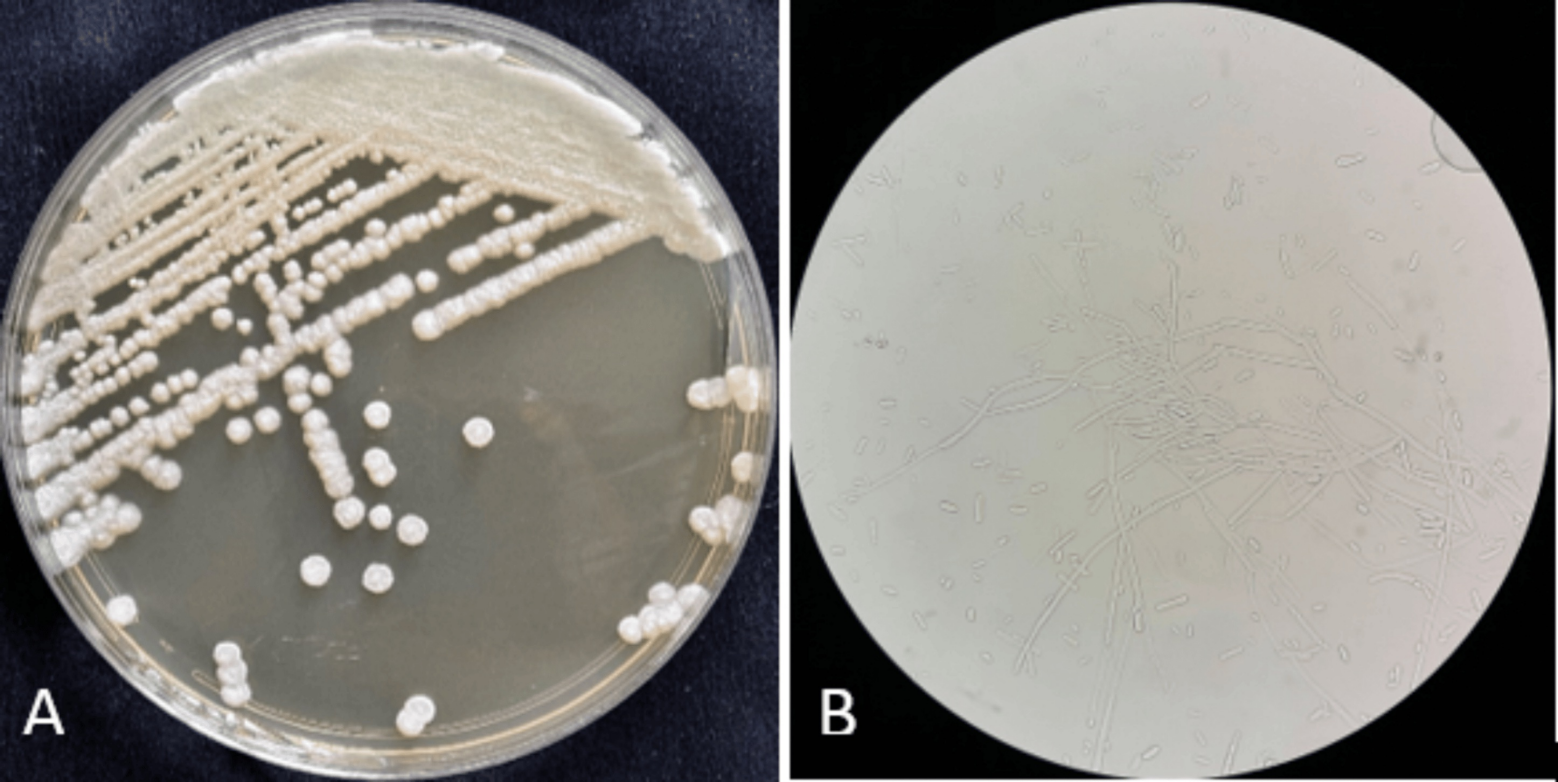
(A) White, dry, cottony, wrinkled, and circular colonies of Saprochaete capitata after 48 hours of incubation of bronchoalveolar lavage (BAL) on Sabouraud chloramphenicol agar. (B) Wet mount preparation of BAL culture showing arthroconidia and septate hyphae with acute-angle branching (400× magnification).

On day three of febrile neutropenia, a BAL was performed. Direct examination revealed septate hyphae and arthroconidia. Smears of the BAL fluid, prepared by cytocentrifugation, were stained with the May-Grünwald-Giemsa method and analyzed under a light microscope using ×40 and ×100 objectives, which helped exclude *Pneumocystis pneumonia*. The galactomannan antigen assay in BAL fluid was negative. The BAL culture yielded results similar to those of the blood culture, isolating *S. capitata* (Figure 4).

Due to the combined antifungal therapy, the patient's clinical symptoms resolved: he became afebrile and eupneic, with normal oxygen saturation, resolution of the cough, and normalization of pulmonary auscultation findings. This clinical improvement was accompanied by a progressive restoration of biological parameters, resulting in the complete normalization of blood count values (white blood cells: 7,680 cells/mm³; neutrophils: 5,400 cells/mm³; hemoglobin: 12.8 g/dL; platelets: 210,000 cells/mm³), as well as inflammatory markers (ESR: 3 mm/h, CRP: 2 mg/L). Serial blood cultures became sterile after the 10th day of therapy.

The patient received a total of one month of antifungal treatment. Clearance of the infection was confirmed through persistent clinical improvement, normalization of laboratory parameters, and negative follow-up blood cultures.

Following resolution of the *S. capitata* infection, the patient continued with consolidation chemotherapy as part of his treatment regimen.

## Discussion

Invasive fungal infections (IFIs) predominantly affect immunocompromised patients, particularly those with hematologic malignancies, agranulocytosis, and recipients of allogeneic hematopoietic stem cell transplantation [6,7]. The incidence of IFIs has considerably increased worldwide during the last decades, making them a major cause of mortality among patients with hematological malignancies [7,8]. *Candida* spp. and *Aspergillus* spp. are the most common causes of IFIs in this setting. However, the emergence of rarer, often antifungal-resistant fungi, such as *S. capitata*, is becoming a significant concern, given the limited therapeutic options available [1,3,4,6].

The taxonomy of *S. capitata* has undergone several revisions since its first identification [9,10]. Various genera names have been used in the literature, such as *Geotrichum* and *Blastoschizomyces* for anamorphs, and *Dipodascus* and *Magnusiomyces* for teleomorphs [2,11]. *S. capitata* is phylogenetically related to ascomycetous yeasts, despite its filamentous thallus lacking budding cells, and is classified in the family *Dipodascaceae*, order *Saccharomycetales* [2,9].

*S. capitata* is an emerging opportunistic fungus responsible for life-threatening infections in immunocompromised patients, primarily those with hematological malignancies and severe neutropenia [5,12]. A literature review conducted by Mazzocato et al. identified 104 cases of systemic infections caused by S. capitata between 1977 and 2013. Hematological malignancies were the most common comorbidities, including acute myeloid leukemia (52%), acute lymphoblastic leukemia (22%), and other hematological malignancies (13%). Non-hematological diseases were observed in 9% of cases, while no comorbidities were reported in the remaining patients. Severe neutropenia was observed in 82% of patients [13]. In our case, the patient, diagnosed with acute myeloid leukemia, presented profound neutropenia at the time of infection, with a neutrophil count of 20/mm³.

The clinical presentation of invasive *S. capitata* infections is atypical, with symptoms resembling disseminated candidiasis and other systemic fungal infections, potentially leading to diagnostic misinterpretation [1,7,14]. Fungemia is the most common clinical form, and deep organ involvement can affect any organ, with a particular predilection for the lungs [7,14]. In our case, the patient presented with invasive *S. capitata* infection, exhibiting fungemia and pulmonary involvement.

The diagnosis of invasive *S. capitata* infection is confirmed through histopathological examination or direct microscopic detection of the fungus from blood or another sterile body site [1]. According to the existing literature, the blood culture positivity rate in *S. capitata* infections can reach up to 70%, while the rates for *Candida *spp. and *Aspergillus* spp. are generally below 50% and 10%, respectively [4,7,14,15]. In a retrospective multicenter clinical study conducted in Italy, 35 cases of *S. capitata* infection were identified, with 74.3% diagnosed via blood culture [16]. In our case, *S. capitata* was isolated from two blood cultures and a BAL sample.

*S. capitata* grows on Sabouraud chloramphenicol agar within 24-48 hours of incubation at 37°C. However, incubation may take up to five days, which may delay the diagnostic process. Macroscopically, the colonies appear white to cream-colored, wrinkled, dry, and cottony, with a frosted glass appearance. Microscopic examination reveals true septate hyphae, pseudohyphae, arthroconidia, and annelloconidia [1,2]. *S. capitata* is thermotolerant, non-fermentative, urease-negative, and capable of growing in the presence of cycloheximide [1,2,6,12].

Some yeasts, including *S. capitata*, are known to possess galactomannan as a component of their cell walls. Cross-reactivity between *S. capitata* and *Aspergillus* galactomannan has been reported in the literature [1,5]. While this cross-reactivity may limit the specificity of the galactomannan test for invasive aspergillosis, awareness of this phenomenon can aid in the diagnosis and management of invasive *S. capitata* infections [1]. In the present case, the galactomannan test was negative.

The treatment of invasive *S. capitata* infections remains challenging. To date, no optimal therapeutic strategy has been established, mainly due to the rarity and diagnostic challenges of this organism, as well as the absence of standardized antifungal breakpoints [1]. According to in vitro susceptibility data from multiple case reports and case series, *S. capitata* is considered intrinsically resistant to echinocandins and exhibits considerable resistance to fluconazole [1,17]. In a previous report studying the in vitro susceptibility of 23 isolates of *S. capitata*, amphotericin B and voriconazole showed the lowest MICs of various antifungal agents, and their combination is the most frequently recommended treatment [18]. The European Society of Clinical Microbiology and Infectious Diseases (ESCMID) and the European Confederation of Medical Mycology (ECMM) guidelines recommend amphotericin B, either alone or in combination with 5-flucytosine or voriconazole, as the most effective therapy for *S. capitata* infections [19]. Amphotericin B, a polyene antifungal, exerts its fungicidal activity by binding to ergosterol in the fungal cell membrane, leading to increased membrane permeability and cell death. Voriconazole, a triazole antifungal, inhibits ergosterol biosynthesis by targeting the fungal cytochrome P450 enzyme lanosterol 14α-demethylase, disrupting membrane integrity and fungal growth [20]. In our case, the patient was treated with intravenous liposomal amphotericin B and voriconazole, based on susceptibility testing and literature guidelines. The patient exhibited favorable clinical and biological responses to treatment.

This case highlights the need for heightened clinical vigilance in immunocompromised patients presenting with febrile neutropenia and respiratory symptoms. *S. capitata*, though rare, should be considered in the differential diagnosis of IFIs, particularly among patients with hematologic malignancies. Early initiation of appropriate antifungal therapy, guided by timely microbiological evidence, is critical for improving patient outcomes. In the absence of standardized treatment guidelines, case reports such as this one play a vital role in informing clinical judgment and guiding therapeutic decisions in similar high-risk clinical settings.

## Conclusions

*S. capitata* is an emerging fungal pathogen responsible for IFIs in severely immunocompromised patients, particularly those with hematological malignancies and profound neutropenia. It presents a significant threat to this vulnerable population due to its high mortality rate. Invasive *S. capitata* infections pose substantial diagnostic and therapeutic challenges, as their clinical presentation is often nonspecific. Additionally, no standardized treatment regimen has been established, and the pathogen exhibits resistance to commonly used antifungal agents, such as echinocandins and fluconazole. Early diagnosis, prompt initiation of appropriate antifungal therapy, careful management of risk factors, and control of underlying conditions are crucial for improving patient outcomes and reducing mortality.
